# Physical Activity Status and Diabetic Retinopathy: A Review

**DOI:** 10.7759/cureus.28238

**Published:** 2022-08-21

**Authors:** Yousif AlQabandi, Savitri Aninditha Nandula, Chinmayi Sree Boddepalli, Sai Dheeraj Gutlapalli, Vamsi Krishna Lavu, Rana Abdelwahab Mohamed Abdelwahab, Ruimin Huang, Shanthi Potla, Sushen Bhalla, Pousette Hamid

**Affiliations:** 1 Ministry of Health, Al Bahar Ophthalmology Center, Sabah Area, KWT; 2 Ophthalmology, California Institute of Behavioral Neurosciences & Psychology, Fairfield, USA; 3 Internal Medicine, California Institute of Behavioral Neurosciences & Psychology, Fairfield, USA; 4 Dermatology, Mansoura University, Mansoura, EGY; 5 Dermatology, California Institute of Behavioral Neurosciences & Psychology, Fairfield, USA; 6 Psychiatry and Behavioral Sciences, California Institute of Behavioral Neurosciences & Psychology, Fairfield, USA; 7 Medicine, Avalon University School of Medicine, Cleveland, USA; 8 Neurology, California Institute of Behavioral Neurosciences & Psychology, Fairfield, USA

**Keywords:** diabetes, risk factors for diabetic retinopathy, sedentary, physical activity level, retina

## Abstract

Substantial evidence highlights the association between physical inactivity and diabetes onset and complications. Little is known regarding the link between physical inactivity and diabetic retinopathy in terms of onset, progression, and severity. This review aims to investigate these associations and understand the underlying mechanisms behind these associations. Decreased sedentary times and the inclusion of more physical activity have been linked to the delayed onset and progression of diabetic retinopathy and less severe forms of said condition. Physical activity provides both protective and anti-inflammatory effects on the retina. Further research is needed to understand and elucidate the exact mechanisms by which lack of physical activity affects retinal health and the onset, progression, and severity of diabetic retinopathy.

## Introduction and background

Physical inactivity remains a significant risk factor for many noncommunicable diseases such as cancer [1], hypertension [2], and diabetes [3], among others. Physical activity has been proven to delay the progression and onset of many of these diseases [4,5]. Some health professionals have included it as an important part of disease prevention and management. Even though there is strong evidence for the correlation between physical activity and disease status, the number of people who actually partake in the minimum requirement for physical activity as prescribed by the American College of Sports of Medicine (ACSM) and other Public Health and Exercise Authorities remains low around the world [6,7]. Physical inactivity remains low on the priority list of many international public health authorities, although it remains a major risk factor for many diseases and conditions. Medication and dietary interventions still outrank physical activity prescriptions. However, physical activity is a relatively lower-cost alternative that could save government spending and is also easily accessible as it can be done from anywhere. Physical inactivity accounts for almost up to 3% of direct healthcare costs [8-11]. 

Physical activity refers to any bodily movement by skeletal muscle that requires energy expenditure, and it can include household chores, sports, and planned exercise, among others [6]. On the other hand, sedentary behavior refers to any sitting activity characterized by minimal energy expenditure and includes television watching, computer use, and reading, among others [6]. Lack of physical activity and sedentary behavior are considered modifiable risk factors for diabetes in general [12]. The correlation between physical inactivity and the onset of diabetes has been established and confirmed to account for almost 27% of all causes of this disease [13]. Physical inactivity is classified as one of the risk factors not only for the onset of type 2 diabetes (T2DM) but also for the progression of said diseases and is a key contributor to many complications such as renal failure, nephropathy, and diabetic retinopathy (DR) [14].

The most common microvascular complication of diabetes is DR which can ultimately lead to blindness as a result. During the first two decades following diagnosis, most type 1 diabetics will develop DR, while almost 60% of type 2 diabetics will be diagnosed with it [14-16]. There are two main forms of DR; they are 1) proliferative diabetic retinopathy (PDR) and 2) nonproliferative diabetic retinopathy (NPDR) [17]. NPDR is the earlier stage of diabetic eye disease and is also the most common form, causing the vision to become blurry [15]. PDR, on the other hand, is a more advanced stage of this diabetic eye disease and occurs when the retina starts to grow new vessels. This form of diabetic eye disease is very serious and can cause macular problems or lead to a detached retina due to the scar tissue that forms because of this disease [15]. 

There is substantial evidence for the association between the progression of diabetes and physical inactivity [18-20], but the correlation between physical activity and the progression of DR needs to be studied further. There are some discrepancies in the findings of studies that looked at the possible correlation between the two as the studies differed in exercise modality and populations studied, among other parameters. Nonetheless, it is necessary to dig deeper into this correlation and understand further the potential insights into this association.

This review aims to investigate the current research to understand the association between physical inactivity and DR. We also aspire to understand if there is an association between the severity of DR.

## Review

Diabetes in general

Physical Inactivity/Sedentary Behavior as a Risk Factor of Diabetic Retinopathy

Physical activity has been studied heavily as an integral part of any diabetes management plan to delay or lessen the severity of diabetes or its complications. Many studies have looked at physical inactivity or sedentary behavior as a risk factor for many diabetes complications, but some have looked at DR specifically and have cited the benefits of partaking in physical activity to delay the onset, and slow progression and severity of DR. Across many parts of the world, researchers have found that physical inactivity has a strong association with diabetic complications, namely DR, by looking at retrospective or collected patient data. For this reason, it is a vital screening tool for DR.

Aro et al. (2019) conducted a study that included 522 individuals to assess if the incorporation of lifestyle changes early in diabetes onset would provide beneficial effects on the occurrence of DR, and they indeed found a positive effect of incorporating lifestyle to diabetes management plans and namely to delay the progression or onset of DR [21]. This study used post hoc analysis by looking at data from the Finnish Diabetes Prevention Study (DPS) and found that those involved in the intervention group of the DPS showed decreased retinal microvascular abnormalities. Keep in mind that the intervention group was subject to a physical activity intervention as well as other interventions, so physical activity is not the only beneficial tool responsible for this result. In agreement with the findings that physical activity could be a potential indicator for DR development and to highlight the importance of screening for DR, Mendoza-Herrera et al. (2017) aimed to develop a screening tool to detect DR early and found that physical activity status is indeed an important tool to include in screening for DR [22]. Wang et al. (2019) conducted the Beijing Eye Study and included 3468 participants in this study and found that higher physical activity was associated with a decreased prevalence of DR [23]. They used a standardized questionnaire and an ophthalmological examination to learn about the physical activity status of individuals.

Bukht et al. (2019) looked at the association between physical activity and diabetic complications among Bangladeshi type 2 diabetic patients [14]. Nine hundred seventy-seven participants were randomly selected from a patient pool in a local hospital, and within this cohort, they found a strong association between physical inactivity and DR in the category they classified as inactive/low physical activity. The association remained high for both the male and female individuals included in their study [14]. At another facility in Bangladesh, Afroz et al. (2019) conducted a retrospective and cross-sectional study in six diabetes hospitals in Bangladesh and included information for 1253 T2DM patients. They found that physical inactivity is a major risk factor associated with microvascular complications, and they recommend the inclusion of physical activity as a vital part of diabetes complications prevention strategies [24]. Alramadan et al. (2019) conducted a survey among 1121 adults with T2DM across three diabetes centers in Saudi Arabia and, using statistical methods, found that inadequate physical activity increased the risk for DR [25]. Yan et al. (2021) conducted a large cohort study involving 9018 working-age diabetic patients to investigate the association between physical activity and DR progression during a 10-year follow-up with said cohort. They found that a higher physical activity level was independently associated with a lower risk of DR progression among this population [26].

Gilbert et al. (2020) looked at specific guidelines for the prevention and management of DR and diabetic eye disease in India and found that the main risk factors for developing said complications included poor control of hyperglycemia and increasing duration of diseases, both of which they stipulated would be hindered by engaging in physical activity as part of lifestyle changes [27]. Yang et al. (2022) included physical activity as a risk factor for DR in diabetic patients when they looked at medical data [28]. Trott, Driscoll, and Pardhan (2022) conducted a review of meta-analyses and found that sedentary behavior was associated with DR risk. They also found a significant protective association between moderate physical activity and DR [29]. Kuwata et al. (2017) found that higher physical activity levels were independently associated with a lower incidence of DR among Japanese patients with T2DM [30]. Khanam et al. (2017) found that among other risk factors, physical inactivity was a significant risk factor for not only DR but also nephropathy and neuropathy [31].

Protective Effect of Exercise on the Retina

Ocular diseases such as DR and age-related macular degeneration result in the degeneration of retinal cells and subsequent loss of vision or blindness [32]. Current treatments are costly and are effective only in certain patients. For this reason, a more convenient and cost-effective treatment is needed. Physical exercise has been postulated to provide a protective effect on retinal health as it has been shown to both lower the risk of age-related macular degeneration and improve visual outcomes [33]. Szalai et al. (2020) coined the term "trained eye," similar to the term "trained heart" used in cardiology to emphasize the beneficial effects of physical exercise on not only heart health but also on the visual system as well [34]. In a review conducted by Li et al. (2019), they further looked at the protective effects of physical activity on retinal health and found promising beneficial effects for this treatment and preventative modality [35]. Ugurlu and Icel (2021) conducted a study to evaluate the effects of physical activity on retinal microvascular structure and included 60 right eyes of 60 individuals splitting the cohort into 30 in the athlete group and 30 in the nonathlete group [36]. They found that in the nonathlete group, there were significantly reduced vessel densities across many retinal microvascular structures [36]. In a review by Bryle et al. (2022), the authors provided evidence from various studies about the benefits of physical activity in reducing the risk of developing DR and stated that less physically active diabetic patients showed increased blood flow in the retina on exertion [37]. In general, the pathogenesis of DR is primarily due to vascular abnormalities in the retina, yet choroidal abnormalities in the eye of patients with DR have also been found [38]. Alten, Eter, and Schmitz (2021) investigated the effects of a four-week high-intensity interval training (HIIT) on choriocapillaris (CC) in type 1 diabetes patients versus healthy young adults. They found that HIIT training was only able to affect CC perfusion in healthy adults but not in diabetic patients, and they postulate that diabetic patients might have impaired CC adaptation [39].

Exercise and Retinal Inflammation

Inflammation plays an important role in the progression and pathogenesis of DR, and at different stages of this condition, chronic low-grade inflammation is perceived [40,41]. The exact mechanism taking place during or after exercise that provides both benefit and protection for the retina remains unknown. One potential mechanism that is postulated to provide this benefit is myokines, with interleukin-6 (IL-6) being the main myokine responsible for this benefit [33]. The myokine IL-6 has both pro- and anti-inflammatory depending on the site and condition they are present in [33,42] and serves more of a pro-inflammatory function in the retina. In patients with DR, aqueous and vitreous levels of IL-6, interleukin-8, vascular endothelial growth factor (VEGF), and chemokine ligand 2 seem to be elevated [40,43]. It is postulated that exercise has the ability to precondition individuals to become less susceptible to chronic inflammation by providing means to combat inflammation. During exercise, IL-6 is released by skeletal muscle and plays more of an anti-inflammatory role by suppressing tumor necrosis factor-α (TNF-α) and interleukin-1β (IL-1β), both pro-inflammatory cytokines [33].

Various studies looked at measuring the effect of physical activity on inflammation. Frith and Loprinzi (2019) included 157 retinopathy patients between 40 and 85 years in their study, where they looked at the effects of moderate-to-vigorous activity and muscle-strengthening exercises on systematic inflammation. They measured inflammation through testing for C-reactive proteins (CRP). They found that moderate-to-vigorous physical activity was inversely associated with systematic inflammation among retinopathy patients, knowing that increased inflammation could increase the severity of retinopathy [44]. They found no association between muscle-strengthening exercises or resistance training and CRP levels. Both Dharmastuti et al. (2018) and Yaribeygi, Butler, and Sahebrak (2019) found that aerobic exercise was confirmed to be a major nonmedical strategy that is both beneficial and protective against the development of major diabetic complications, including DR [45,46]. The molecular effects they cited for aerobic exercise included the inhibition of inflammatory responses and oxidative stress. They also found that prolonged daily sedentary behavior was closely associated with a higher incidence of retinal dysfunction in diabetic patients.

Several studies looked at animal models to understand the pro- or anti-inflammatory benefits of exercise on retinal health. In line with previous research [42,47], treadmill exercise illustrated that in a streptozotocin-induced model of DR, exercise was able to inhibit neuronal cell death and suppress VEGF expression in the retina after exercise [48]. In another animal model of obese rats, de Campos et al. (2020) looked at short-term combined aerobic and resistance exercise on inflammatory profile in the retina. They utilized treadmill exercise and climbing movements and found that combined exercise was able to reverse the elevated levels of pro-inflammatory proteins (TNF-α and IL-1β) in the serum and the retina independently of changes in adiposity in the body [49].

Exercise and the Severity of DR

Large amounts of studies have shown that physical inactivity is a risk factor for DR, but the question of whether or not physical inactivity has an association with the severity of DR remains unclear. Several studies have looked at that relationship. Praidou et al. (2017) observed the correlation between physical activity and DR and found that increased physical activity was associated with less severe levels of DR [12]. The cohort included in their study were 240 patients with T2DM consisting of 80 patients with mild-to-moderate NPDR, 80 patients with severe to very severe NPDR, and 80 with PDR and compared with 80 nondiabetic patients who served as the control group. Shen et al. (2020) conducted the Jiangsu Diabetic Eye Disease Study, in which they investigated the prevalence, causes, and risk factors of moderate or severe visual impairment and blindness in people with T2DM over 50 years old in Funing County, Yancheng, China. Across 85 survey sites, they included 1909 participants in their investigation, and they found that DR was the second leading cause of blindness and visual impairment after cataracts. Blindness and moderate-to-severe visual impairment were high within the studied population for both conditions, and they cited many risk factors, which included physical inactivity [50]. Frith and Loprinzi et al. (2018) investigated the impact of nonbouted, or lifestyle versus bouted, or structured form of physical activity on retinopathy prevalence and its severity among a selected cohort. They found that those who participated in lifestyle physical activity had reduced odds of having a retinopathy diagnosis, and those who engaged in less physical activity had more advanced or more severe forms of DR [51]. Tikkanen-Dolenc et al. (2020) found that frequent leisure-time physical activity was associated with a lower incidence of severe DR in type 1 diabetes [52]. Similarly, Hassabi et al. (2020) evaluated the association between physical activity and the severity of DR and found that lower physical activity levels are identified as an independent risk factor for more severe and higher stages of DR in contrast to those who engaged in moderate physical activity [53]. They suggest that physical activity lowers the risk of DR progression by lowering the body mass index (BMI) and achieving better glycemic control. Finally, Ren et al. (2019) conducted a review of current studies that looked at the association between physical activity or lack of and risk of DR. They found that in diabetic patients, physical activity had a protective association with DR and found this influence stronger on vision-threatening DR or on more severe forms of DR [54,55].

## Conclusions

The studies highlighted in this review ascertain the positive association between physical activity and both the onset and severity of DR; however, the exact type and frequency of physical activity are yet to be defined. Currently, there is no definite proof that the same exercises that have a protective effect on the retina are the same between type 1 and type 2 diabetes, but we postulate the same mechanisms exist between the two types, and physical activity is ultimately beneficial in both cases. A limitation of this review was the fact that we highlighted research documenting the benefits of varying physical activity for DR progression, onset, and severity, but the data were not fully able to create a direct correlation between physical activity and the latter. Based on these data, we were not able to create a definite exercise program that can create the positive impact from exercise that we aspire to with DR patients. We also focused on articles that showed a positive correlation between physical activity and DR and did not highlight any that showed a detrimental impact of physical activity on DR, although we have not seen any such articles. Future research should compare varying exercise modalities, and longer longitudinal studies need to be elaborated. Studies with larger populations as well as those that look at various inflammatory factors are necessary to fully understand the mechanisms responsible for the onset, progression, and severity of DR.
